# Multispecies Benchmark Analysis for LC-MS/MS Validation and Performance Evaluation in Bottom-Up Proteomics

**DOI:** 10.1021/acs.jproteome.3c00531

**Published:** 2024-01-20

**Authors:** Tobias Jumel, Andrej Shevchenko

**Affiliations:** Max Planck Institute of Molecular Cell Biology and Genetics (MPI-CBG), Pfotenhauerstraße 108, 01307 Dresden, Germany

**Keywords:** data-independent acquisition (DIA), label-free quantification (LFQ), benchmark, DIA-NN, accuracy, differential proteomics analysis, LC-MS/MS

## Abstract

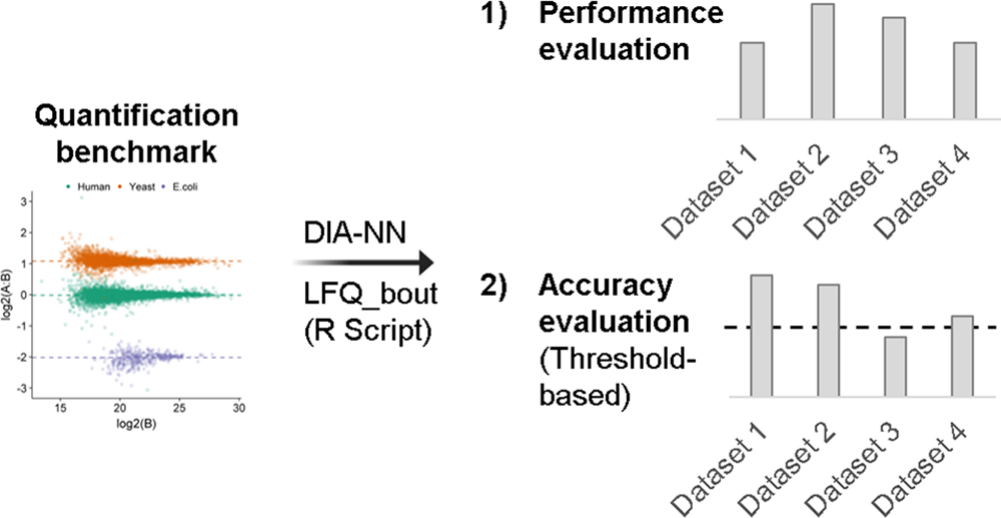

We present an instrument-independent benchmark procedure and software (LFQ_bout) for the validation and comparative evaluation of the performance of LC-MS/MS and data processing workflows in bottom-up proteomics. The procedure enables a back-to-back comparison of common and emerging workflows, *e.g*., diaPASEF or ScanningSWATH, and evaluates the impact of arbitrary and inadequately documented settings or black-box data processing algorithms. It enhances the overall performance and quantification accuracy by recognizing and reporting common quantification errors.

## Introduction

Data-independent acquisition (DIA)^1^ is becoming increasingly popular in bottom-up proteomics as specialized instrumentation and software become readily available (reviewed in refs (2−4)). The performance and quantification accuracy of DIA workflows are typically benchmarked using mixtures of total proteome digests from 2 to 4 species, as first described by Kuharev et al. and later introduced as the LFQbench package by Navarro et al.^5,6^ Analyzing the series of samples with predefined fold changes between individual proteomes allows comprehensive assessment of the quantification accuracy at the proteome-wide scale.^5−15^

However, we have noticed that the output of benchmarking experiments is typically presented as protein group - level scatter or density graphs together with a single summary statistics related to the difference between expected and measured fold changes. While it lacks the appropriate summary statistics and thresholds, it is difficult to detect errors and to perform consistent evaluation of the quantification accuracy and compare workflows performance.

To streamline evaluation and optimization of workflows, we combined the protein quantification with the R script LFQ_bout that provides summary statistics and supports visualization of benchmark results. The procedure includes differential analysis of the abundance of protein groups similar to previously described protocols.^9,14^ Benchmarking typically relies on comparative quantification of proteome mixtures of defined but varying composition. In this context, mixing total protein digests from different species emulates proteome-wide changes; however, the magnitude of these changes is exactly known. We underscore that benchmarking cannot rely on monitoring the abundance of a few selected proteins but ideally should be having a proteome-wide coverage.

We demonstrate that the impact of this straightforward, yet comprehensive benchmark analysis extends beyond routine quality control and is critical for setting the field standard of integrity, interlaboratory consistency, and interpretation transparency of quantitative proteomics experiments.

## Experimental Procedures

### Data and Code Availability

The benchmark analysis script and supporting materials are available at https://github.com/t-jumel/LFQb. Raw data are available at MassIVE-KB (https://massive.ucsd.edu/ProteoSAFe/static/massive.jsp) with the identifiers MSV000090837 and MSV000090832 (Figures S1 and S2). External raw data reanalyzed in this work (not from QE-HF instrument) were from PXD028735 (https://proteomecentral.proteomexchange.org/cgi/GetDataset?ID=PXD028735).

### Benchmark Samples and Experiment Design

Multispecies sample mixtures^5^ with expected log2 fold changes (A/B) of 0 for human, +1 for yeast, and −2 for *E. coli* were prepared using Pierce HeLa Protein Digest Standard (Thermo Fisher Scientific), MS Compatible Yeast Protein Extract, Digest (Promega GmbH, Walldorf, Germany), and MassPREP *E. coli* Digest Standard (Waters Corporation, Milford, USA). 100 μg of each digest were dissolved in 555.5 μL of 0.2% formic acid, yielding the expected peptide concentration of 0.18 μg/μL. Mixing these stocks in volumetric ratios (human: yeast: *E. coli*) of 65:30:5 yields Sample A and 65:15:20 yields Sample B, both with the total peptide concentration of 0.18 μg/μL. Injecting 5 μL of either Sample A or Sample B resulted in a total load of 0.9 μg on the column that was equivalent of 45 ng *E. coli*, 270 ng yeast and 585 ng human protein digests for Sample A and 180 ng *E. coli*, 135 ng yeast, and 585 ng human protein digests for Sample B. The analysis was performed in triplicate in block randomized fashion.^16^ In general, the benchmark procedure and supporting R-script (see below) are compatible with samples of any composition containing digests of 2–4 different species mixed in defined ratios.

The normalization benchmark data were acquired using 1 μg sample A on column and 0.7 μg sample B on column in 2 technical replicates using 30 min elution gradients by both DDA and DIA methods in a block–randomized fashion. The diaPASEF and other non-QE-HF raw data were from PXD028735.^15^

### LC-MS

The LC-MS setup (Thermo Fisher Scientific, except where indicated) consisted of an UltiMate 3000 UHPLC system equipped with an Acclaim PepMap precolumn (100 μm × 20 mm, C18, 5 μm, 100 Å) and a 50 cm μPAC pillar array column. Five μL samples were injected at a flow rate of 5 μL/min and eluted using a 2-sloped linear 90 min gradient delivered at the flow rate of 0.5 μL/min from 0 to 17.5% ACN in 60 min (two-thirds of the gradient length) and 17.5–35% ACN in 30 min (one-third of the gradient length) in 0.1% FA. The LC was coupled to a QE-HF hybrid mass spectrometer via a μPAC Flex iON interface plus nESI emitter (20 μm, 5 cm, Fossiliontech, Madrid, Spain). Electrospray voltage was 2.5 kV, transfer capillary temperature was 280 °C, and S-lens RF level was 50%.

### Proteomics Data Acquisition and DDA Raw Data Processing

Data-independent acquisition (DIA) method consisted of a full MS scan (*m*/*z* range of 395–955; *R*_*m*/*z* 200_ 30,000; 3 × 10^6^ automatic gain control; 55 ms injection time; centroid mode) followed by 31 MS2 scans under *R*_*m*/*z* 200_ 30,000; 1 × 10^6^ automatic gain control (AGC); 55 ms injection time; centroid mode; width of the isolation window *m*/*z* 18; normalized collision energy (NCE) 24%; fixed first mass *m*/*z* 100; acquisition range of *m*/*z* 400–950 for precursor and *m*/*z* 100–2000 for fragment ions after demultiplexing with staggered DIA windows.^17^ Raw files were demultiplexed and converted to mzML using MSConvert v3.0.2^18^ and processed with DIA-NN v1.8, using predicted spectral libraries.^7^ Visualization of DIA data was performed in Skyline-daily v21.2.1.514.^19^

Data-dependent acquisition (DDA) method included MS1 scan (*m*/*z* range of 350–1700; *R*_*m*/*z* 200_ 60,000; 3 × 10^6^ AGC; 55 ms injection time; profile mode) followed by Top15 data-dependent MS2 (*R*_*m*/*z* 200_ 15,000; AGC 1 × 10^5^; 50 ms injection time; centroid mode; width of isolation window *m*/*z* 1.6 with *m*/*z* offset of +0.2; NCE 24%; fixed first mass *m*/*z* 100; dynamic exclusion 20 s; all charges excluded except 2–5). DDA data were analyzed with MaxQuant v1.6.17.0^20^ and MSFragger v3.1.1/FragPipe version 14.0^21^ with default settings without match between runs (MBR) option.

### DIA Raw Data Processing

The “default” settings of DIA-NN v1.8 were adjusted to ensure that the MBR is disabled. Precursor and fragment *m*/*z* ranges for data acquired on QE HF were *m*/*z* 400–950 and *m*/*z* 100–2000, respectively. “Optimized” DIA-NN settings include --cut K*,R* --var.-mods 1 --var.-mod UniMod:35,15.994915,M --double-search --individual-mass-acc --individual-windows --smart-profiling --pg-level 2 --species-genes --peak-center --no-ifs-removal --no-quant-files --report-lib-info --il-eq --matrix-qvalue 0.005 --nn-single-seq For database searches, we used canonical Swiss-Prot subsets of the UniProt reference proteomes as of 20.08.2021 (human - UP000005640, yeast - UP000002311, and *E. coli* - UP000000625) as well as the MaxQuant contaminant database.^20^ Raw data from PXD028735^15^ were analyzed with optimized DIA-NN analysis settings as provided above and with precursor and fragment *m*/*z* ranges of *m*/*z* 399–1201 and *m*/*z* 50–2000 for diaPASEF and TTOF5600, *m*/*z* 399–901 and *m*/*z* 50–2000 for QE-HF-X, Scanning SWATH, as well as *m*/*z* 399–1201 and *m*/*z* 100–1500 for TTOF6600Swath.

### Benchmark Analysis Script

The DIA-NN protein group and precursor matrices were analyzed with our in-house developed R script LFQ_bout available together with the example of input and output data sets at https://github.com/t-jumel/LFQb. The protein group matrix was employed for benchmark tests: as compared to the unique genes matrix, it could be error-prone because of potential inclusion of nonprototypic peptides. Upon execution, the script reported an average and median CV, asymmetry factors, confusion matrix summary statistics, and statistics related to the log2-fold change values. Entries matching common MaxQuant contaminants were removed since no ground-truth values of log2 fold changes could be assigned. Entries were considered as identified if data completeness exceeded 50%, i.e., a value was reported in at least 2 out of 3 replicates in both Samples A and B. Identified entries having a coefficient of variation (CV) below 20% in both Samples A and B were considered as quantified.

The asymmetry factor^22^ was derived from the density function of log2 fold change values by dividing the left and right distances between the center line and the *x*-values at 10% of the maximum height. An asymmetry factor <1 indicated underestimation of fold changes and a value >1 indicated overestimation. Thresholds for reaching an undesirable degree were 0.5 and 2, respectively.

The abundances of quantified protein groups between the samples were compared using Limma v3.50.0 based on log2-transformed intensities and robust empirical Bayes statistics. Protein groups with BH-adjusted p-values of less than 0.01 and log2 fold change exceeding ±0.5 with no upper limit were classified as “Up” or “Down”, respectively. By comparing these measurements with expected composition of sample mixtures, we obtained confusion matrix summary statistics.^23^ The approach was similar to that used in refs (9 and 14). Our focus was on the number of true positives (TP) and the false discovery rate, here called deFDR to differentiate it from the precursor and protein group identification FDR. True positives also included protein groups whose values of log2 fold change vastly exceeded the values expected from sample composition. This inaccuracy was not recorded with confusion matrix statistics but with quantification-related statistics such as the “Accuracy” summary statistic as described below.

The deFDR was calculated according to the confusion matrix as FP/(FP + TP). It is a single value per each benchmark and serves as a summary indicator of identification errors. While workflows can be optimized to reduce the deFDR, the value itself and the related true positives count are not adjusted to a fixed value (e.g., 1%), but compared against a predefined threshold–in contrast to the protein group identification FDR. Other confusion matrix statistics included the sensitivity or true positive rate calculated as TP/ (FP + TP), and the specificity or true negative rate calculated as TN/ (TN + FP).

As additional indicators, we also introduced secondary summary statistics. The “Accuracy” statistic referred to the average distance between expected and measured log2-fold changes and was not associated with confusion matrix counts. The “Dispersion” was the average distance between measured log2-fold changes and their respective medians. The “Trueness” was the sum of the distances between measured median and expected log2 fold changes for the species involved.

## Results

### Common Limitations of Current Benchmark Procedures

**Figure 1:**
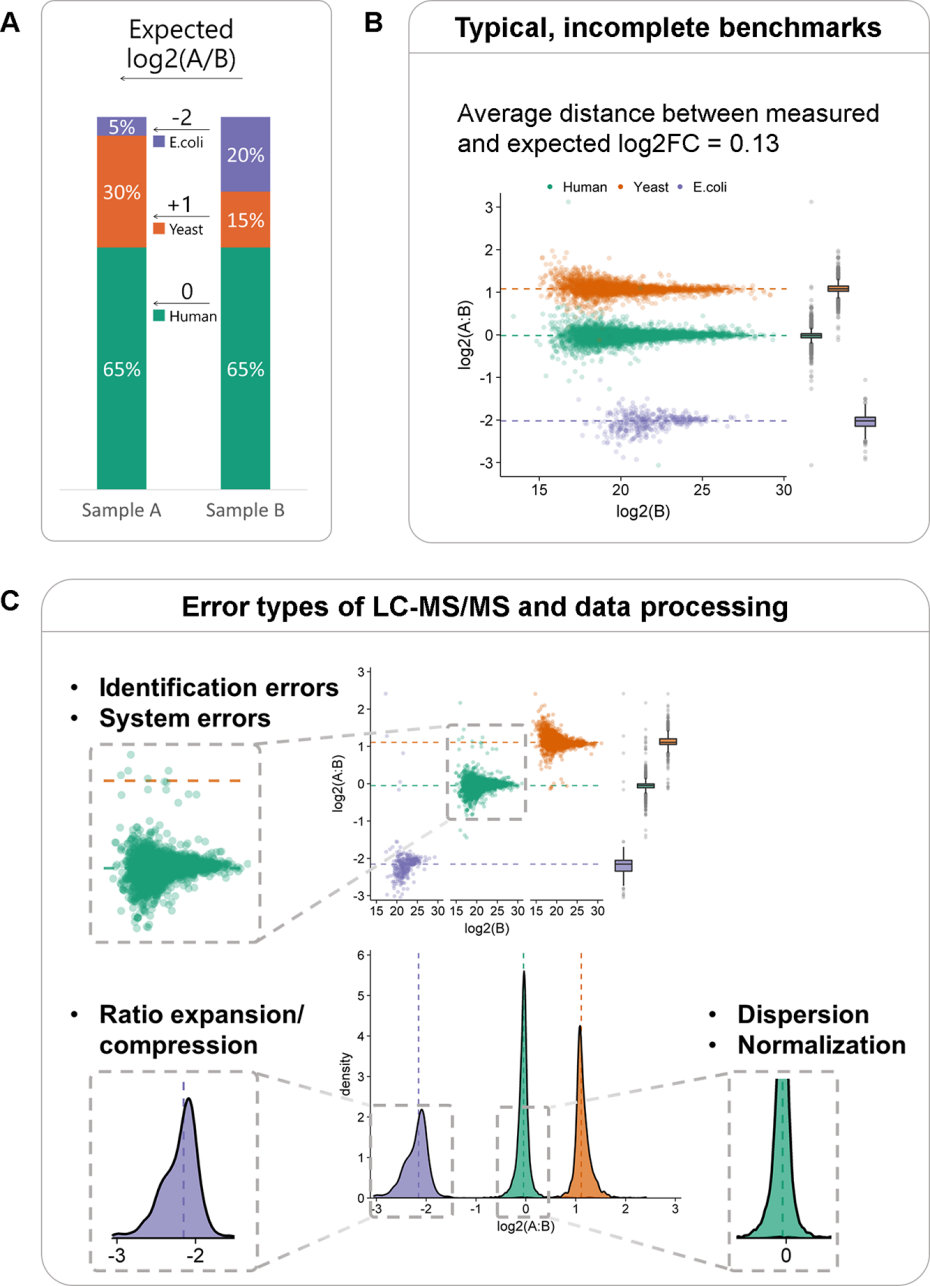
Limitations of current bottom-up proteomics benchmark protocols. (A) Multispecies sample types used for QE-HF measurements and data from PXD0287352. (B) Benchmarking typically reports scatter plots and differences between expected and measured fold change values of protein groups, providing limited insight into data quality. (C) Typical types of errors that benchmarks should be able to detect, as exemplified by QE-HF DIA data analyzed with default settings in DIA-NN v1.8. Facet and density plots illustrate major error types that should be revealed using the summary statistics.

Quantification accuracy in untargeted, label-free, bottom-up proteomics is not rigorously defined. While typical multispecies benchmarks provide ad hoc estimate of the accuracy, we argue that common reporting formats are insufficiently informative (Figure 1A,B). In particular, the use of a single scatterplot and a single summary statistic of the distance between measured and expected log2-fold changes does not elaborate on different types of errors. Based on our experience and extensive benchmarks, including reprocessing raw data from repositories,^15^ we have identified five major sources of errors in bottom-up proteomics (Figure 1C). They are as follows:

(i) incorrect identifications resulting from mismatched peptide sequences and/or compromised precursor and protein group identification cutoffs; (ii) quantitative dispersion, typically expressed as coefficient of variation (CV). Note that low dispersion/high precision is a prerequisite for high accuracy; (iii) nonsystematic over- or underestimation of fold changes. Here, expansion and compression are collectively termed as distortion; (iv) system errors leading to erroneous peak intensities in the raw data (Figure S4); (v) cross-run normalization errors during data processing (Figures S1 and S2).

**Figure 2:**
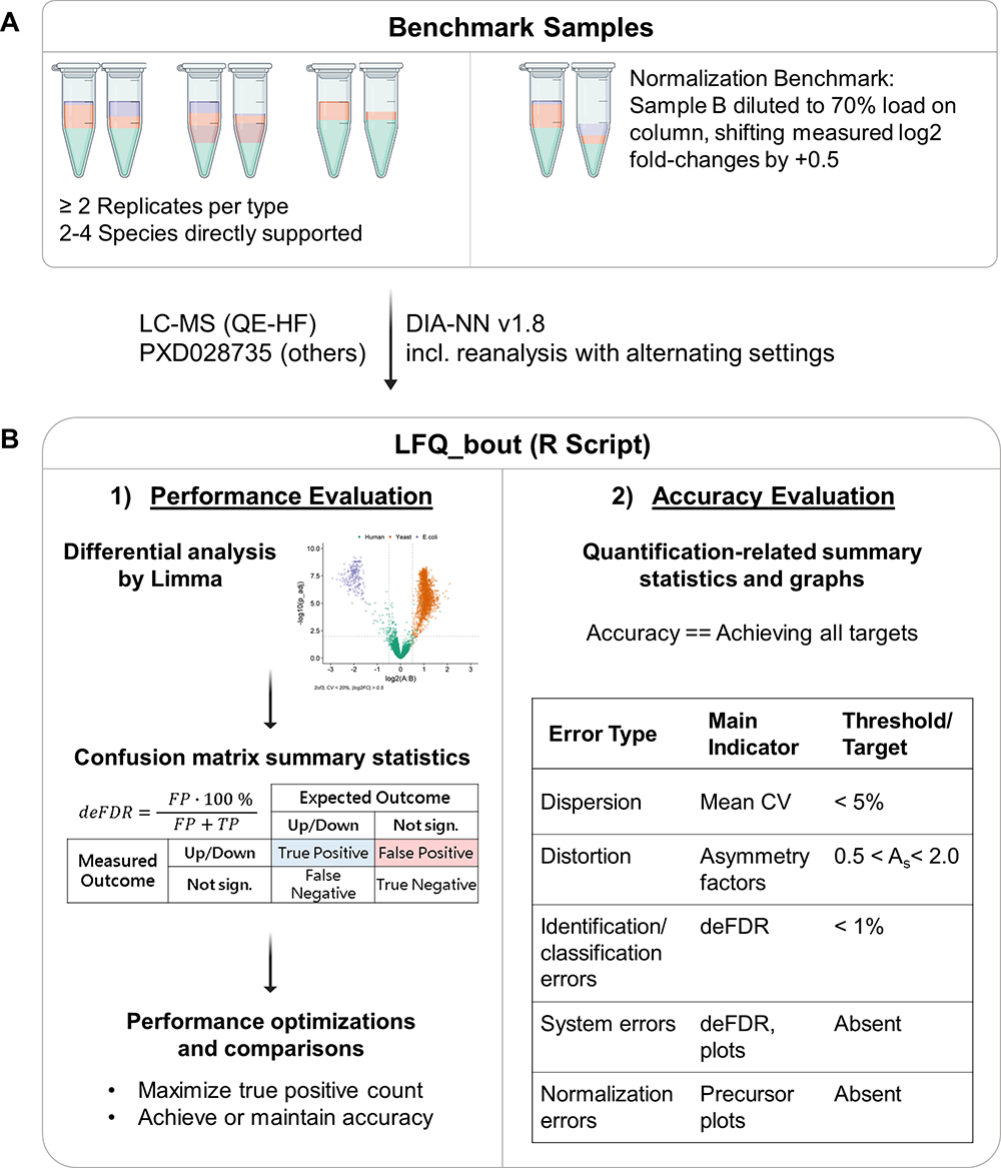
Multifaceted benchmarks for validation and evaluation of untargeted bottom-up proteomics workflows. (A) R script processes data acquired from all benchmark samples. Analysis of a diluted sample is required for validating the data normalization. (B1) Quantified protein groups are subjected to differential analysis that provides summary statistics for evaluating workflow performance. (B2) Overview of minimal summary statistics and threshold values. Only workflows that met these criteria are termed accurate. Workflow performance is evaluated by the true positives count.

We reasoned that multispecies benchmarks should be used to eliminate system and normalization errors, and to recognize and limit errors related to dispersion, distortion, and identification (Figure 2B2). Errors were evaluated (Figure 2) by our R script LFQ_bout, which defined the examined workflow as either accurate or inaccurate. Instead of only monitoring ID numbers, LFQ_bout evaluated and compared workflow performance using the number of true positives and precisely quantified protein groups.

The benchmark workflow relies on differential abundance analysis of protein groups. Based on the measured fold changes and adjusted p-values, protein groups were classified as “Up”, “Unchanged”, or “Down” and compared with the trends expected from the composition of analyzed protein mixtures. In this way, each protein group quantification is further classified as true (or false) positive (TP/FP) or true (or false) negative (TN/FN).

The number of protein groups correctly assigned as “Up” (TP count) was used as the main workflow performance indicator, together with the quantification accuracy evaluation. The TP count was always accompanied by the classification error rate/differential analysis false discovery rate (deFDR = FP/(FP + TP)). The deFDR and related graphs indicated if the examined workflow led to an increased rate of mismatches because of poor identification stringency and/or whether classification errors were increased because of high ratio expansion or error-prone normalization. If a workflow exceeded the 1% deFDR threshold, it was termed inaccurate. Other summary statistics describing dispersion and distortion (Figures 1 and 2) were derived directly from the measured log2 fold changes of protein groups. The graphical output produced for both protein groups and precursors also detected unexpected systematic and normalization errors that could only be recognized at the precursor level.

We proposed that comparative ranking of workflows could rely on true positive counts; however, only TP counts having deFDR values below the threshold are meaningful. Workflow optimization should aim at maximizing the TP count while meeting all accuracy requirements outlined in Figure 2B2.

### Benchmark-Guided Workflow Evaluation and Optimization

We exemplified the value of our benchmark procedure by comparing QE-HF data processed under different DIA-NN settings. This test case is practically relevant, and it can be used as a guidance for other workflows optimization.

We compared QE-HF data processed four times under different DIA-NN settings that are supposed to control how chromatographic peak boundaries are recognized and peaks integrated. However, their impact on the performance and accuracy of the workflow was unknown. In total, we tested four combinations of “high precision” or “high accuracy” analysis modes together with “robust LC” or “any LC” settings. The DIA-NN user manual indicated that the “high accuracy” mode performs additional interference subtraction, while under the “robust LC” settings the software does not integrate tails of chromatographic peaks.

**Figure 3:**
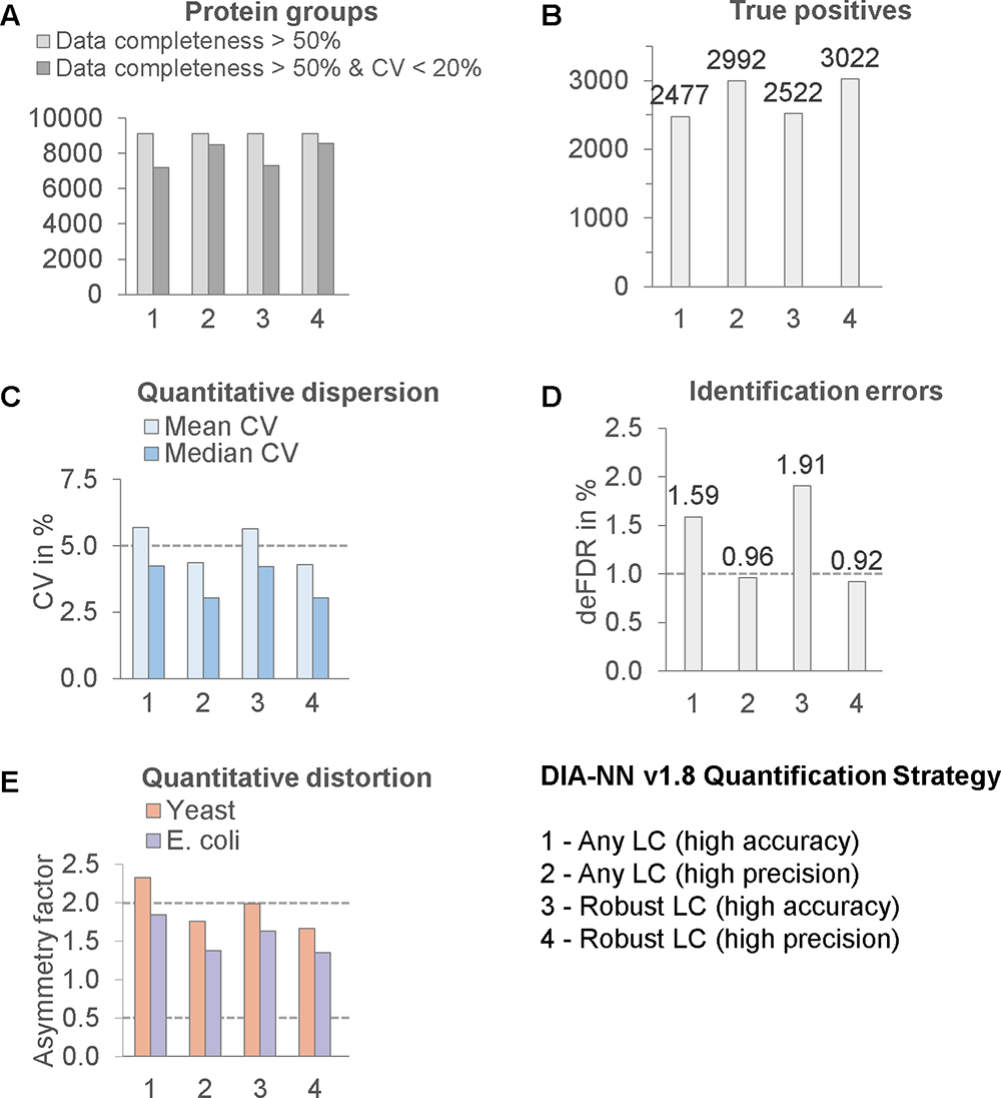
Benchmark-guided evaluation and optimization of LC-MS workflows. DIA data from QE-HF were analyzed with DIA-NN v1.8 using 4 quantification strategies with unknown influence on quantitative accuracy. As shown in panels C, D, and E, result sets 1 and 3 were not accurate, but 2 and 4 were accurate. Using “high accuracy” mode increased classification error, worsened precision, and increased ratio expansion. Result sets 2 and 4 were of acceptable level of error and higher proportion of the proteome was accurately quantified (CV < 20%) as shown in panel A. This translated into higher true positive values (panel B). Overall, the “high precision” settings improved both the performance and the accuracy of quantification.

We observed that the performance differences between “any LC” and “robust LC” modes were rather small (Figure 3), but there was a critical performance gap between the “high precision” and “high accuracy” modes. Furthermore, both “high accuracy” workflows were, in fact, inaccurate due to increased standard deviations (mean CV > 5%), increased classification error (deFDR > 1%), and increased distortion. The “high precision” modes also resulted in higher true positive counts and performance due to the higher number of protein groups passing 20% CV cutoff filter.

In particular, the “high precision” mode combined with the “robust LC” mode was found to be accurate and best performing, while the most commonly used default setting (“any LC” and “high accuracy”) reduced performance and resulted in inaccurate quantification. The optional removal of interference from the “high accuracy” modes did not improve the analyses, while the drawbacks, such as reduced precision, resulted in a net negative impact on quantitative accuracy.

**Figure 4:**
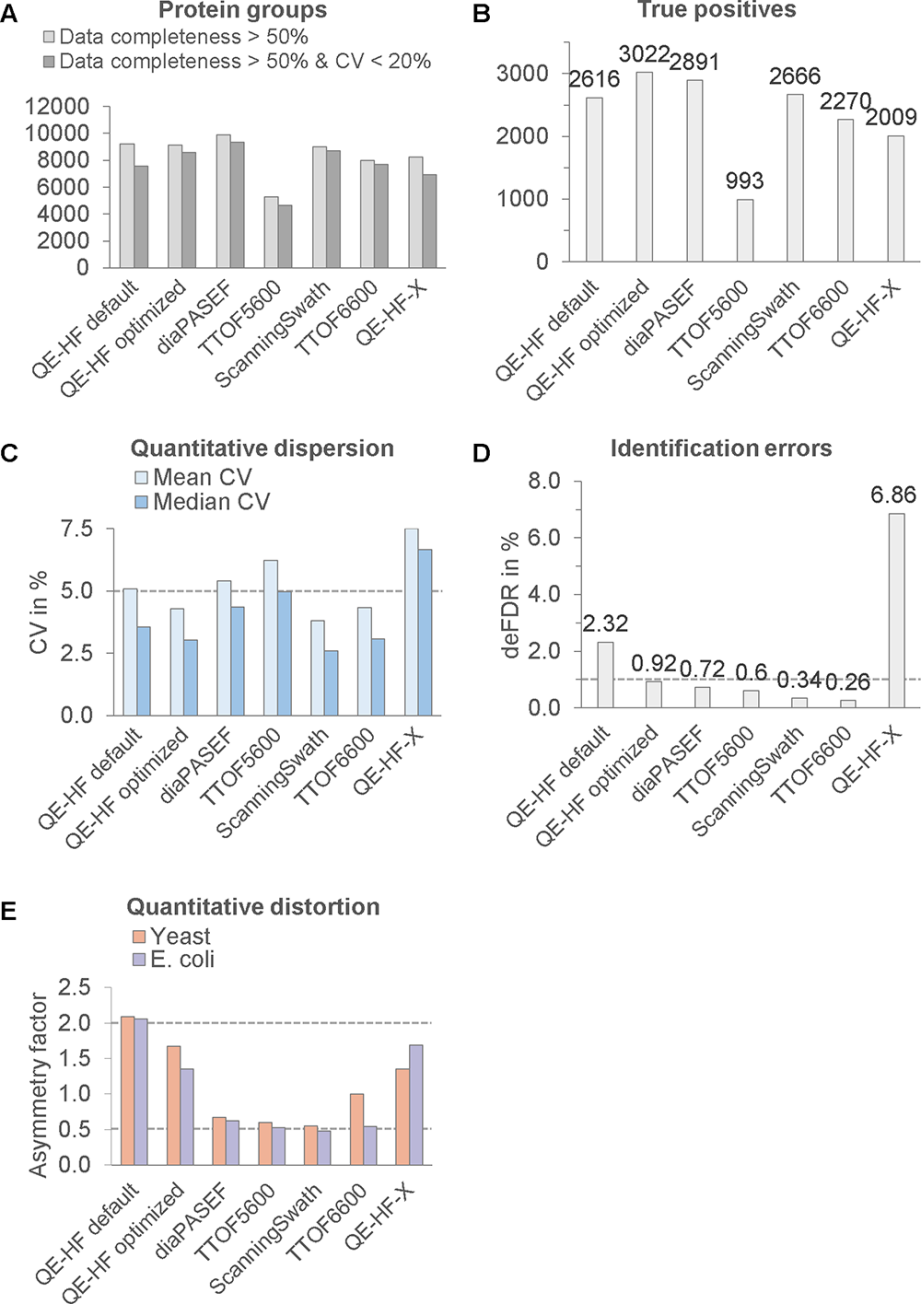
Improved cross-platform performance and accuracy assessment. QE-HF DIA data were obtained with 0.9 μg on column loading, 90 min gradient elution, and both default and optimized DIA-NN v1.8 settings (A–E). Other results are from raw data of PXD028735 obtained with 1.0 or 5.0 μg loaded on the column and 120 min elution gradients, analyzed with DIA-NN settings optimized for QE-HF data (A–E). Dashed lines represent thresholds that must not be exceeded for a data set to be termed accurate.

Our benchmark protocol was further used to determine the impact of all relevant DIA-NN settings on the QE-HF data, similar to the case study above. Processing of QE-HF data under default DIA-NN settings lacked accuracy (Figure 4C–E). Two rounds of stepwise optimization were performed to maximize the number of TPs while maintaining high accuracy (Table S2).

Changing the quantification strategy to “robust LC” and “high precision”, together with the double-pass mode and protein group identification with FDR of 0.5%, was beneficial. The results of the QE-HF optimized workflow (Figure 4) passed all thresholds and outperformed default DIA-NN settings in all quality and performance aspects.

### Cross-Platform Performance Evaluation

Next, we extended the benchmark scope to comparing the results acquired on different instrumentation platforms. The data set PXD028735^15^ offered the samples with multispecies protein extracts, which we reanalyzed under DIA-NN settings optimized as described above. These results have inherent advantages over the QE-HF data due to longer elution gradient (120 min vs 90 min), higher sample loads (1.0 or 5.0 μg vs 0.9 μg), and using DIA-NN double pass mode, which is beneficial, yet not typical for diaPASEF and other large collections of raw data.

The analyses of five additional data sets are shown in Figure 4. Only the TTOF 6600 SWATH data were as accurate as optimized QE-HF data (Figure 2), yet they did not outperformed them.

In particular, the results from QE-HF-X were found to be inaccurate, mainly due to deFDR exceeding 6% (Figure 4D), based on the underlying system error shown in Figure S4. This error was characterized by a distorted distribution of fragments intensity at the lower tail of the distribution, and for unknown reason, a subset of precursors was quantified with drastically shifted log2 fold changes. This error could be easily overlooked if only basic graphical reporting and statistics were employed. These issues were specifically addressed by LFQ_bout.

The other result sets acquired on hybrid instruments with ToF mass analyzers were prone to significant underestimation of fold changes (ratio compression), as judged by asymmetry factors close to 0.5 (Figure 4E). This ratio compression could be a reason for the relatively low classification error rate having deFDR below 1% (Figure 4D).

However, due to the ratio compression and higher standard deviation (Figure 4A,C) of multiple result sets such as the diaPASEF, their performance ranking according to the number of true positives would be lower than the ranking according to ID numbers (Figure 4A,B). The optimized QE-HF workflow achieved the highest performance because it was less affected by the above limitations despite slightly lower ID numbers as compared to diaPASEF.

## Discussion

We have developed a benchmarking strategy and software that provide a comprehensive and practical assessment of the quantification accuracy and performance of bottom-up proteomics workflows. Our protocol is based on multispecies benchmarking analyses using confusion matrix summary statistics.^9,14^ It covers cross-run normalization algorithms, identification/classification errors, quantitative dispersion, and distortion. In addition, unusual and unexpected errors such as system error in the QE-HF-X data and ratio expansion in the QE-HF data were successfully detected.

Our procedures revealed that for QE-HF data, the default DIA-NN settings lead to suboptimal performance and inaccurate quantification. This is to be expected as DIA-NN was designed for processing data acquired using short LC gradients on hybrid ToF mass spectrometers. In contrast, hybrid QE HF instruments offer very different characteristics in terms of chemical noise and peak interference in chromatograms (Figure S3) that make default settings suboptimal. Our benchmarking procedure provides a tool for efficient optimization of LC-MS/MS workflows, regardless of how well the software features or unfamiliar components are documented or understood by end-users.

The inclusion of true positive counts as a performance indicator provides a more accurate ranking of workflow performance than ID numbers by incorporating fold change over- or underestimation as well as the quantification precision. The asymmetry factors representing the distortions may be an oversimplification of complex mathematical distributions, but it is important to highlight the associated trends in a practical way with a number that subjectively matches the visual data representations. Other summary statistics and nonlogarithmic fold changes were uninformative to describe the distortion, yet they are still included in a separate script output table. While the ratio compression in the ToF data was expected, the dominant ratio expansion in the Orbitrap was unexpected, and we may have uncovered a potential risk factor compromising consistency across different instrument platforms.

Overall, we were pleasantly surprised by the insights and performance gains achieved by benchmark-guided optimization. We were also able to show that even older mass spectrometers without ion mobility separation can achieve similar performance as modern mass spectrometers when using 90 min or longer elution gradients of. Furthermore, we could show that using the DIA-NN protein group matrix instead of the unique genes matrix is a viable option to achieve accurate quantification without being restricted to prototypic peptides if the DIA-NN data processing is carefully validated and optimized.

Another useful feature of this benchmark method is that it supports comparative evaluation of the quantification performance across different LC, MS, and software platforms often running under poorly defined settings. While now (and, likely, in a foreseeable future) proteomics is (and will) remain cross-platform, being able to compare the analytical performance in a stringent and unbiased way is an important step toward better analytical consistency and standardization, which is particularly important for translational and clinical applications.

The main limitation of our benchmark method is that it does not account for errors related to biological variability and sample preparation. Therefore, it may be beneficial to replace the CV threshold of the accuracy criteria with an application-relevant optimization of sample preparation, chromatography, and MS scan speed to maximize the number of proteins quantified with CV below 20%.

Extensive use of our proposed summary statistics on modern instruments may be required to refine the empirical thresholds, especially for deFDR applied to ToF data. However, this work clearly demonstrates the potential of the proposed benchmark principles for workflow validation and optimization.

## Conclusions and Perspectives

Benchmarks are essential tools to establish accurate and comprehensive quantification in bottom-up proteomics. However, they remain undervalued due to the lack of software and rational rules of interpretation. While other *omics* disciplines are establishing GSP (good scientific practice) standardization,^24−26^ we feel it should also become routine in proteomics especially because computational methods might have a decisive, but poorly understood impact on the results quality. Our benchmark procedure allows proteomics practitioners to better understand the impact of computational algorithms, even without being able to access the code or understand their mathematical background. We feel that simple and rational benchmark procedures provide impactful information about the overall quality and validity of protein quantification workflows. The proteomics community will greatly benefit from adopting this protocol.

## Supporting Information Available

Benchmark variant to validate cross-run normalization in bottom-up proteomics by LC-MS; results of the validation of normalization algorithms in bottom-up proteomics by LC-MS; manual assessment of quantitative distortions; improved benchmarking procedure also allows the detection of unexpected system failures; benchmark summary statistics of QE-HF data analyzed with optimized DIA-NN v1.8 settings while again cycling through DIA-NN quantification strategies; and benchmark summary statistics of QE-HF data analyzed with default DIA-NN v1.8 settings and QE-HF and PXD0287352 data analyzed with optimized DIA-NN settings (PDF)
